# Maternal separation modifies spontaneous synaptic activity in the infralimbic cortex of stress-resilient male rats

**DOI:** 10.1371/journal.pone.0294151

**Published:** 2023-11-09

**Authors:** Jesús David Ayala-Rodríguez, Jesús García-Colunga

**Affiliations:** Departamento de Neurobiología Celular y Molecular, Instituto de Neurobiología, Universidad Nacional Autónoma de México, Juriquilla, Querétaro, México; University of Illinois at Urbana-Champaign, UNITED STATES

## Abstract

Glutamate and GABA signaling systems are necessary to maintain proper function of the central nervous system through excitation/inhibition (E/I) balance. Alteration of this balance in the medial prefrontal cortex (mPFC), as an effect of early-life stress, may lead to the development of anxiety and depressive disorders. Few studies exist in the infralimbic division of the mPFC to understand the effect of early-life stress at different ages, which is the purpose of the present work. Newborn Sprague Dawley male rats were subjected to maternal separation (MS) for two weeks. First, tests measuring anxiety- and depression-like behaviors were performed on adolescent and adult rats subjected to MS (MS-rats). Then, to establish a relationship with behavioral results, electrophysiological recordings were performed in neurons of the infralimbic cortex in acute brain slices of infant, adolescent, and adult rats. In the behavioral tests, there were no significant differences in MS-rats compared to control rats at any age. Moreover, MS had no effect on the passive membrane properties nor neuronal excitability in the infralimbic cortex, whereas spontaneous synaptic activity in infralimbic neurons was altered. The frequency of spontaneous glutamatergic synaptic events increased in infant MS-rats, whereas in adolescent MS-rats both the frequency and the amplitude of spontaneous GABAergic events increased without any effect on glutamatergic synaptic responses. In adult MS-rats, these two parameters decreased in spontaneous GABAergic synaptic events, whereas only the frequency of glutamatergic events decreased. These data suggest that rats subjected to MS did not exhibit behavioral changes and presented an age-dependent E/I imbalance in the infralimbic cortex, possibly due to differential changes in neurotransmitter release and/or receptor expression.

## Introduction

Early-life stress (ELS) is a key factor for the development of psychiatric diseases such as anxiety and depression [1, 2]. Infancy is characterized by many developmental changes that, when they are altered, impact importantly on behavior later in life [3–5]. Furthermore, in animals and humans, ELS produces morphological, molecular, and biochemical changes in many brain regions, such as the hippocampus and prefrontal cortex, which are related to anxiety and depression [6–13].

Due to its late development, the medial prefrontal cortex (mPFC) is a brain structure broadly affected by ELS [14]. For instance, in male adolescent rats stressed by maternal separation (MS-rats), a well-known animal model of ELS, the spine density on apical and basal dendrites decreases in mPFC pyramidal neurons and the expression of GluR1 and GluR2 α-amino-3-hydroxy-5-methyl-4-isoxazolepropionic acid receptor (AMPAR) subunits increases, resulting in impairment of long-term potentiation [6]. However, contradictory structural and behavioral effects have been described in adult rats [15, 16], suggesting that these changes are not long-lasting but rather plastic, and that the animals may show a resilient profile after MS. In this regard, adolescent MS-rodents showed neither anxiety- nor depressive-like behaviors, even with prolonged MS [17, 18]. This lack of responses may be associated with resilience to ELS. Furthermore, rodents subjected to mild and moderate MS presented proactive behaviors, indicative of stress resilience [16, 19, 20]. On the other hand, the mPFC of adolescent male MS-rats loses interneurons, an effect regulated by the activity of *N*-methyl-*D-*aspartate receptors expressing the subunit NR2A [7], indicating an excitation/inhibition (E/I) imbalance related with anxiety-like behavior. In the same sense, it has been described that in prelimbic pyramidal neurons of MS-rats, the E/I balance and intrinsic excitability are altered in an age-dependent manner, showing in adolescent rats a greater disinhibition while in adult rats the opposite [13]. Nevertheless, to our knowledge, there are no reports of changes in the E/I balance in the infralimbic cortex of resilient rats subjected to MS. Recently, it was described that exposition to chronic stress during adolescence prevents the reduction of neuronal excitability in infralimbic neurons of rats exposed to a prolonged stress in adulthood; additionally, these rats showed less fear conditioning compared to rats not exposed to the ELS [21].

In the present study, we performed a battery of tests for exploring anxiety- and depressive-like behaviors in rats subjected to MS. In addition, whole-cell patch-clamp recordings were performed to examine the impact of MS on both intrinsic excitability and synaptic activity in infralimbic pyramidal neurons. This work was conducted in male rats at three developmental stages: infancy, adolescence, and adulthood. We used the combination of these techniques to determine changes in the infralimbic E/I balance in MS-rats and their relationship to resilience and/or psychiatric-like behaviors.

## Materials and methods

All experimental procedures were carried out in accordance with the National Institute of Health Guide for Care and Use of Laboratory Animals and were approved by the Institutional Animal Care Committee of the Universidad Nacional Autónoma de México, with an effort to minimize the number of animals used and their suffering.

### Animals

Pregnant female Sprague Dawley rats (2 weeks pregnancy) were received from the animal housing facility of the Instituto de Neurobiología of the Universidad Nacional Autónoma de México and were maintained in a standard laboratory cage under controlled environment (24 ± 2° C; 12 h light-dark cycle) with *ad libitum* food and water. Dams were acclimated to the new room for at least 5 days and randomly assigned into control or MS groups. Dams were observed twice per day (9:00 and 17:00 h) to confirm delivery. The putative day of birth was designated as postnatal day zero (P0) and the next day (P1) the MS protocol started after adjusting the litter size to eight pups (four males and four females).

### Maternal separation

The MS protocol, with minor changes, was performed as previously reported [6]. From P1 to P15, pups of the MS group were carefully placed in a new cage bedded with woodchips for 4 h (10:00–14:00 h) next to the home cage containing the dam; after this time the pups were carefully returned to the home cage. Home cages were cleaned twice per week after previously subjecting the rats to MS, and control animals were always manipulated in the same way as MS-rats but without being separated from the dam. On P22, all animals were weaned and housed 2–4 per cage from the same litter, group, and sex. Only male rats were used in this project.

### Body and adrenal weights

Body and adrenal weights are indirect biomarkers of the impact of stress [22, 23]. Body weight gain was measured at P16, P22, P32 and P60. The adrenal glands were dissected from rats of P16, P32 and P62, soaked in distilled water and dried with paper towel. Most of the glands were collected from rats used in electrophysiological experiments. Adrenal weight data correspond to the ratio of adrenal weight relative to the body weight of the rat (mg/g). Just one rat per litter was used to compare the adrenal weight, so each data represents one litter. For the case of the MS effect on body weight, 1–4 rats per litter were used; when more than one rat was used from a litter, the weights were averaged. All measurements were collected between 10:00–12:00 h to avoid circadian effects.

### Behavioral tests

All rats were manipulated for at least 5 days before behavioral tests. The tests were performed between 9:00–12:00 h in an experimental room where the rats were habituated for at least 1 h, except the sucrose preference test (SPT), which was done in the housing room. Just adolescent and adult rats were used for behavioral tests. Only one rat per litter was used in each test, except in the forced swimming test (FST) for adolescents and the SPT for both ages, where two rats per litter were used. In these later cases, the result of the two rats were averaged to represent their respective litter to avoid any litter effect [24–26]. Each apparatus was cleaned with 70% alcohol after being used with each rat. The experimenter was not present in the room during the tests, except for the splash test (ST), in which animals were habituated to the experimenter for at least 15 minutes. Blind experimenters analyzed all data, except those of the open field test (OFT), where analysis was automated.

#### Open field test

**Fig 1:**
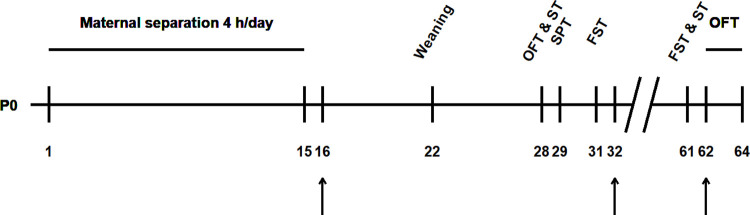
Protocol of MS. Experimental timeline of MS protocol, behavioral tests and electrophysiological recordings (indicated by arrows).

Locomotor activity and anxiety-like behavior in a novel environment were assessed with OFT for both adolescent (P28) and adult (P62-64) rats (Fig 1). To record the activity of animals, one batch of rats from each group was placed for 15 minutes in the center of an acrylic box (42 x 42 x 39 cm) with a grid of photobeam sensors in the floor [27]. The total distance traveled and the time in the center were analyzed with Fusion SuperFlex Software (Omnitech Electronics, Inc., OH, USA).

#### Forced swim test

We used the modified version of the FST [28]. Adolescent rats started the test at P30 and adult rats at P60. The test was divided into two sessions: the first session was a habituation pre-test in which rats were placed, for 15 minutes, inside a glass cylinder (45 x 15 cm) filled with water (30 cm depth) at 24–26°C. At the end of this session the rats were gently dried with a paper towel; the water was changed after every session. The second session was done for 5 minutes 24 h after the pre-test, with everything performed similarly as the pre-test; here, the rats were placed into the cylinder in different order than in the previous day. On both days, swimming activities of the rats were recorded for 5 minutes with a video camera; the first 5 minutes of the pre-test was recorded. Swimming, climbing, and immobility behaviors were assessed off-line in bins of 5 seconds, with a total of 60 counts each session. The predominant behavior in each bin was chosen for the analysis [29]. Because immobility is indicative of behavioral hopelessness, only this was used for statistical analysis.

#### Splash test

This test assesses the motivation to engage in self-care [30–32]. Adolescents (P28) and adults (P61) rats were sprayed, while they were in their home cages, with a 10% sucrose solution on the dorsal coat and recorded for 5 minutes. The latency to the first self-grooming and percent of time spent grooming were evaluated.

#### Sucrose preference test

For this test, the rats were first habituated three days to the presence of two bottles filled with water, one on each side of the cage lid. Then, one bottle was filled with a 1% sucrose solution [33] for four days. The position of the bottles was switched every 24 h to avoid position preference, and the sucrose solution was changed every 48 h. After this adaptation, rats were water deprived for 24 h, and the test was performed the next morning after weighing the rats: at P30 for adolescent and at P61 for adult rats (Fig 1). Each rat was placed individually in a new cage with two pre-weighed bottles, put carefully on each side, one with water and one with sucrose solution. The test lasted one hour, and the bottles were switched each 30 minutes. At the end of the test, the bottles were carefully removed and weighted. The percent of sucrose preference was calculated according to the formula:

%sucrosepreference=(sucrosesolutionconsumed/(sucrosesoltionconsumed+waterconsumed))×100

With water and sucrose solution consumption divided first by the weight of the rats [34]. Drinking less sucrose solution indicates the presence of anhedonia.

### Electrophysiological recordings

#### Slices preparation

Infant and adolescent rats were deeply anesthetized with isoflurane, perfused with ice cold (4°C) solution containing (in mM): 250 sucrose, 2.5 KCl, 1.2 NaH_2_PO_4_, 5 MgCl_2_, 0.5 CaCl_2_, 26 NaHCO_3_, and 10 glucose, pH 7.4, and then decapitated. Their brains were quickly removed and placed into the same cold solution. Coronal slices (350 μm thick) containing the mPFC [35] were cut from individual rats with a Vibratome Leica VT 1000S and submerged in artificial cerebrospinal fluid (ACSF) containing (in mM): 125 NaCl, 2.5 KCl, 1.23 NaH_2_PO_4_, 1 MgCl_2_, 2 CaCl_2_, 26 NaHCO_3_, and 10 glucose (pH 7.4). The slices were stabilized in ACSF for at least 1 h before electrical recording while continuously bubbled with 95% O_2_ and 5% CO_2_ at room temperature. For adult rats, they were anesthetized, decapitated, and the brains removed and placed into the same solution as above. Coronal slices containing the mPFC were cut (300 μm thick) and submerged in ACSF continuously bubbled, as with the other ages, with the difference that slices were first incubated during 30 min in the solution heated ~34°C, and the rest of the time at room temperature.

#### Whole cell patch-clamp recordings

Individual slices were transferred into a tissue chamber and superfused throughout the experiment with ACSF at a rate of 2–3 ml/min. Individual neurons were visualized using an infrared video-microscopy system (BX51WI, Olympus Instruments, Japan) endowed with an 80x water immersion objective. Infralimbic pyramidal neurons from the layers V-VI were recognized based on their large somata localized at least 500 μm from the pial surface [36]. Whole cell voltage-clamp recordings [37] were performed with a PC-ONE Patch/Whole Cell Clamp (Dagan Corporation, MN, USA), using a Digidata 1440A acquisition system controlled by pClamp10 software. Patch-clamp electrodes were made with borosilicate glass (Sutter Instrument, CA, USA) having a resistance of 7–12 MΩ when filled with an internal solution composed of (in mM): 140, KCl, 5, NaCl, 1 MgCl_2_, 10 HEPES, 10 EDTA (adjusted pH7.4 with KOH). Experimental data were stored in a computer using a Digidata 1440A AD converter (Axon Instruments, Union City, CA, USA) at a sampling rate of 10 kHz. The spontaneous inhibitory postsynaptic currents (sIPSCs) and excitatory postsynaptic currents (sEPSCs) were recorded in cells held at -70 mV, in the presence of the AMPAR antagonist 6-cyano-7-nitroquinoxaline-2,3-dione (CNQX, 10 μM) and the GABA_A_ receptor (GABA_A_R) antagonist bicuculine (10 μM), respectively. After 10 minutes of a baseline recording, the antagonists were added to the ACSF at least 10 minutes [38]. The amplitude and the frequency of sIPSCs and sEPSCs were analyzed off-line with Mini Analysis 6.0.3. (Synaptosoft) with a threshold set at 8 pA.

Under current-clamp mode, 200-ms pulses were applied to infralimbic neurons, ranging from -250 to +250 pA with 50 pA increments. Resting membrane potential was recorded from neurons at the moment of breaking the membrane. The passive membrane properties and intrinsic excitability were analyzed as follows. Input resistance was evaluated from the slope of the current-voltage (I/V) relationship in the range of hyperpolarized currents (i.e., from -250 to 0 pA). Phase plots of d*V*/d*t* as a function of membrane voltage were constructed from the first action potential generated with a depolarized current. Then, threshold of the action potential (AP) was obtained, defined as four times the standard deviation of d*V*/d*t* from baseline. The afterhyperpolarization (AHP) amplitude was calculated from the difference between the absolute values of the most negative point from the ellipse of the plot and the threshold, and the maximum and minimum d*V*/d*t* [39]. The AP amplitude was calculated from the voltage value between the threshold and the maximum of the AP; half width was defined as the duration at half AP amplitude. Number of AP spikes were counted in each depolarizing current step. Instantaneous frequency is defined as the inverse of the inter-spike interval (ISI) between the two first spikes generated by a depolarized step. The ISI ratio was evaluated as the last ISI divided by the first ISI, from depolarized pulses that evoked at least five APs. The sag ratio was obtained from the response to the maximum hyperpolarizing current step, as the difference between the minimum voltage and the final voltage at the offset of the step, and this divided by the minimum voltage [40]:

(Vmin−Vfinal)/Vmin.

#### Statistical analysis

All data except for synaptic activity were compared with linear mixed-effects models, because the groups were not balanced [41, 42]. A Kolmogorov-Smirnov 2-samples test with a bootstrap of 2000 iterations was used to compare the cumulative distributions of the interevent interval (IEI) and amplitude of spontaneous postsynaptic currents [43–45]. The quantal size (*q*) was calculated from amplitudes of sIPSCs by fitting a Gaussian distribution [46, 47]. To compare *q* values, a weighted t-test was used. *p* < 0.05 was considered statistically significant for the linear mixed-effects models comparisons and the weighted t-test, while *p* < 0.01 was considered statistically significant for the Kolmogorov-Smirnov test. The results are expressed as mean ± SEM. All statistical comparisons were calculated with R software (v. 4.1.0, R Foundation for Statistical Computing, Vienna, Austria). We used the package *lmerTest* v. 3.1.3 for the linear mixed-effects models, the *F* values were calculated with the package *sjstats* v. 0.18.1, and the weighted t-test was conducted using the package *weights* v. 1.0.4.

## Results

### Body and relative adrenal weights

To study the effects of MS on different developmental stages, we measured both the body and relative adrenal weights in infant, adolescent, and adult male rats. As expected, there were significant age-dependent increases of both body and adrenal weights (S1 Fig); however, MS had no effect on these weights.

### Behavioral test battery

**Fig 2:**
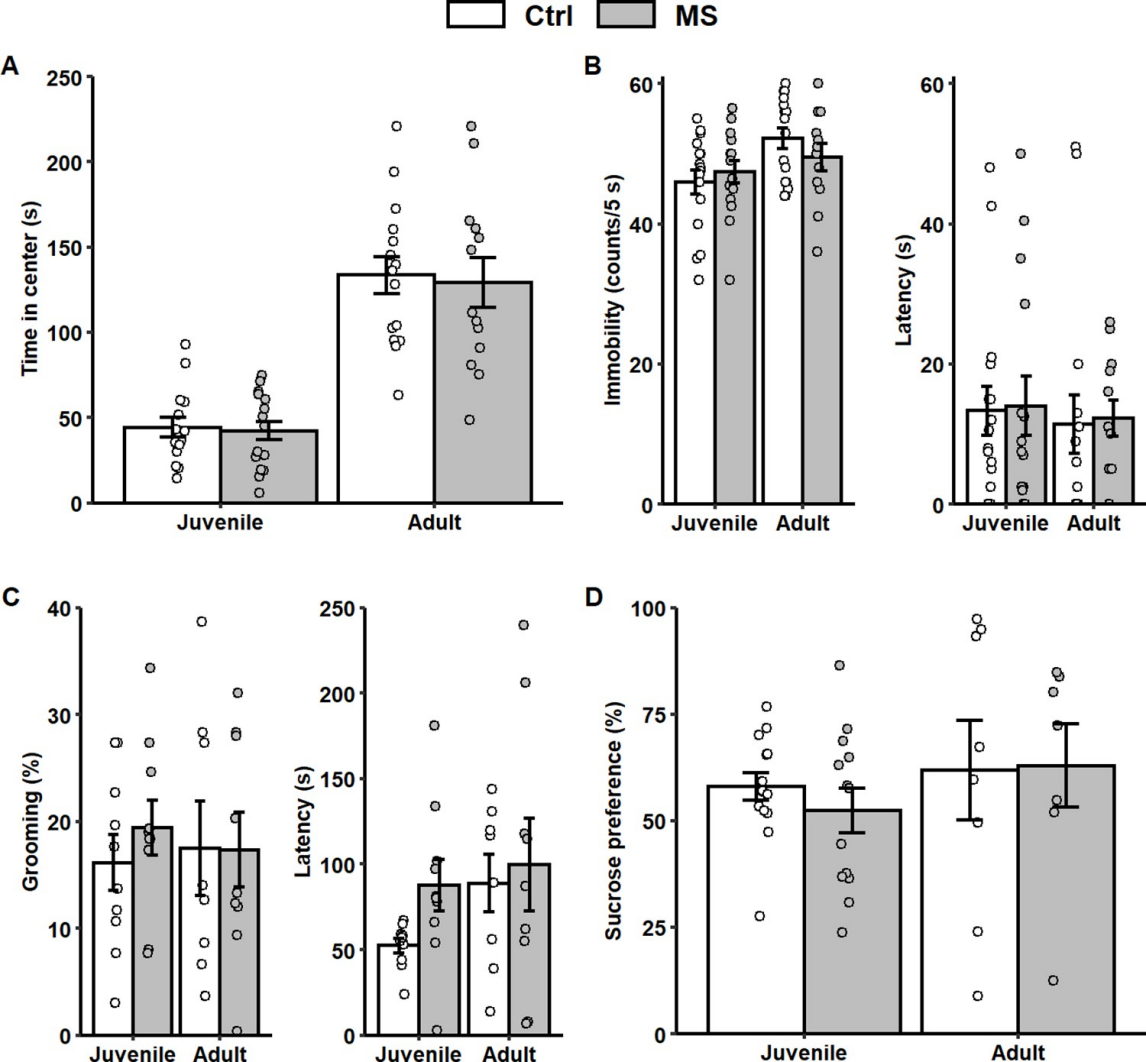
Performance of juvenile and adult rats in behavioral tests. **A.** Time in the center of the open field (number of rats: juvenile control/MS = 15/16; adult control/MS = 15/13, linear mixed-effects models [group: F(1,43) = 0.189, *p* = 0.67, age x group: F(1,43) = 0.42, *p* = 0.84]). **B.** Counts of immobility (left, linear mixed-effects models [group: F(1,41) = 0.2, age x group: F(1,42) = 1.96]) and time of latency to first immobility (right, linear mixed-effects models [group: F(1,41) = 0.11, *p* = 0.74, age x group: F(1,42) = 0.02, *p* = 0.89]) in the FST (number of rats: juvenile control/MS = 16/15; adult control/MS = 16/12). **C.** Percent of time grooming (left, linear mixed-effects models [group: F(1,25) = 0.18, *p* = 0.68, age x group: F(1,25) = 0.5, *p* = 0.49) and time of latency to the first grooming (right, linear mixed-effects models [group: F(1,26) = 2.43, *p* = 0.13, age x group: F(1,26) = 0.6, *p* = 0.45) in the ST (number of rats: juvenile control/MS = 10/10; adult control/MS = 8/9). **D.** Percentage of sucrose intake in the SPT (number of rats: juvenile control/MS = 14/13; adult control/MS = 8/7, linear mixed-effects models [group: F(1,19) = 0.02, *p* = 0.89, age x group: F(1,19) = 1.68, *p* = 0.21). Data represent means ± SEM. Each dot represents each rat.

MS is an animal model for studying both depressive- and anxiety-like behaviors after a constant ELS [6, 12, 18]. To rule out the possible contribution of developmental alterations due to early experimental interventions to rats, the total distance traveled and water intake were measured with the OFT and SPT, respectively. There were no differences between juvenile and adult rats in the total distance traveled in the OFT (S2 Fig) nor total water consumption in the SPT (S3 Fig). After this, we tested whether MS induced anxiety-like behaviors in the OFT (Fig 2A). There was no difference for the time spent in the center. Similarly, there were no differences in depressive-like behaviors when MS-rats were assessed with FST, ST and SPT (Fig 2). Taken together, these results indicate that MS was not associated with enhanced anxiety or depressive-like behaviors.

### Synaptic activity

**Fig 3:**
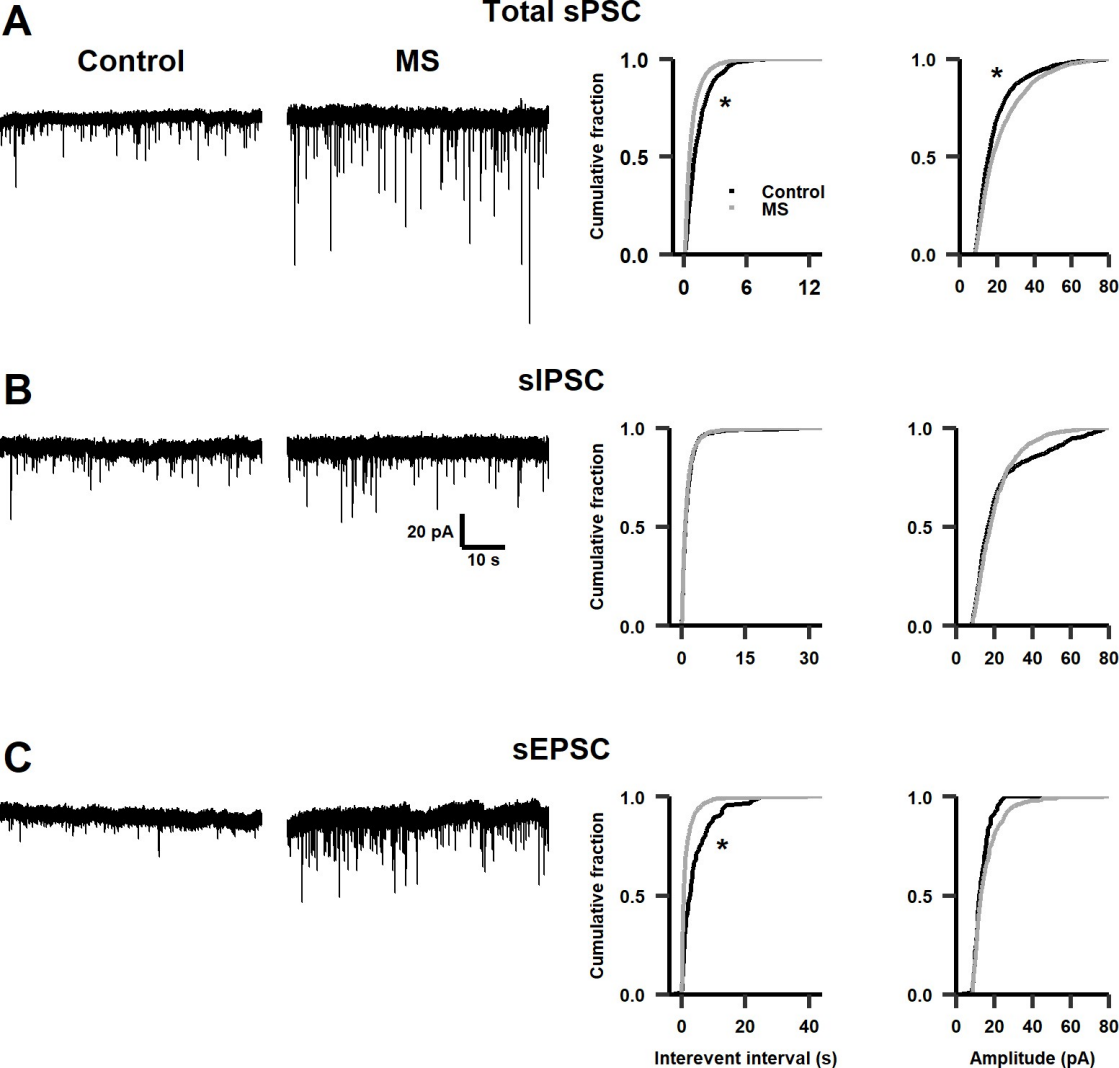
Effects of MS on synaptic activity in infant rats. **A.** Left panel: representative traces of total sPSCs in infralimbic cortex of control and infant MS-rats. Right panel: cumulative distribution plots of the interevent interval (Kolmogorov-Smirnov test, D = 0.27703, *p* < 0.001) and amplitude (Kolmogorov-Smirnov test, D = 0.13273, *p* < 0.001) of sPSCs (n = 12–14 cells, N = 7–10 rats). **B.** Similar as in (**A**) for sIPSCs (n = 7 cells, N = 3–6 rats, interevent interval Kolmogorov-Smirnov test, D = 0.06301, *p* = 0.2, amplitude Kolmogorov-Smirnov test D = 0.08974, *p* = 0.013). **C.** Similarly as in (**A**) for sEPSCs (n = 5–6 cells, N = 5–6 rats, interevent interval Kolmogorov-Smirnov test, D = 0.3609, *p* < 0.001, amplitude Kolmogorov-Smirnov test, D = 0.15775, *p* = 0.017). * *p* < 0.01.

To explore whether MS causes electrophysiological changes in the infralimbic cortex, synaptic activity in pyramidal neurons was recorded under whole-cell voltage-clamp configuration in infant, adolescent, and adult male rats. First, in infralimbic neurons of infant MS-rats (P16), the IEI of spontaneous postsynaptic currents (sPSCs) decreased, which corresponds to a leftward shift of the cumulative distribution of IEI of sPSCs, i.e., an increased frequency of synaptic events (Fig 3A); and the amplitudes of sPSCs increased, observed as a rightward shift of the cumulative distribution of amplitudes of sPSCs (Fig 3A). To determine if these differences were due to changes in inhibitory or excitatory synaptic activity, both sIPSCs and sEPSCs were pharmacologically isolated (see Materials and Methods). Nevertheless, we found no significant differences in either the frequency or amplitude of sIPSCs (Fig 3B). On the other hand, the IEI of sEPSCs decreased significantly in MS-rats, with no significant differences in their amplitude (Fig 3C). These results indicate that glutamatergic synaptic activity increased in the infralimbic cortex of infant rats as a consequence of MS.

**Fig 4:**
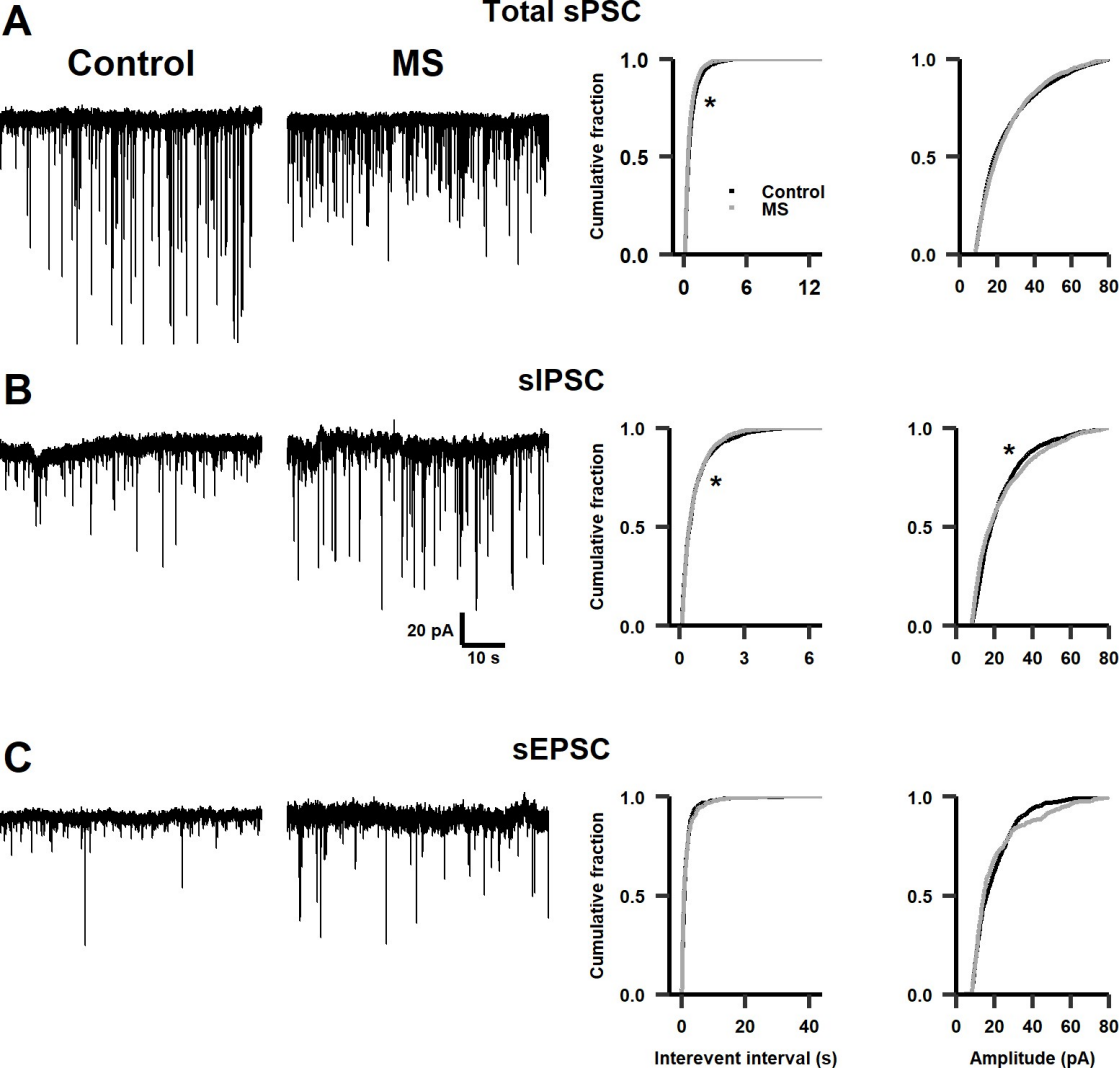
Effects of MS on synaptic activity in adolescent rats. **A.** Left panel: representative traces of total sPSCs in infralimbic cortex of control and adolescent MS-rats. Right panel: cumulative distribution plots of the interevent interval (Kolmogorov-Smirnov test, D = 0.041343, *p* < 0.01) and amplitude (Kolmogorov-Smirnov test, D = 0.038669, *p* = 0.01) of sPSCs (n = 13–16 cells, N = 10–13 rats). **B.** Similar as in (**A**) for sIPSCs (n = 8 cells, N = 6–8 rats, interevent interval Kolmogorov-Smirnov test, D = 0.060167, *p* < 0.01, amplitude Kolmogorov-Smirnov test D = 0.089126, *p* < 0.001). **C.** Similar as in (**A**) for sEPSCs (n = 5–8 cells, N = 5 rats, interevent interval Kolmogorov-Smirnov test, D = 0.084334, *p* = 0.09, amplitude Kolmogorov-Smirnov test D = 0.088304, *p* = 0.054). * *p* < 0.01.

Moreover, the IEI of sPSCs decreased in infralimbic neurons of adolescent MS-rats, without modifying their amplitudes (Fig 4A). After isolating sIPSCs, we also found a decrease in the IEI of sIPSCs in neurons of adolescent MS-rats, corresponding to an increase in the frequency of sIPSCs; however, the amplitude of sIPSCs increased (Fig 4B). There were no significant differences in either the frequency or amplitude of sEPSCs (Fig 4C). Interestingly, these data show an increased inhibition of the infralimbic cortex in adolescent MS-rats, different from that found in infant MS-rats.

**Fig 5:**
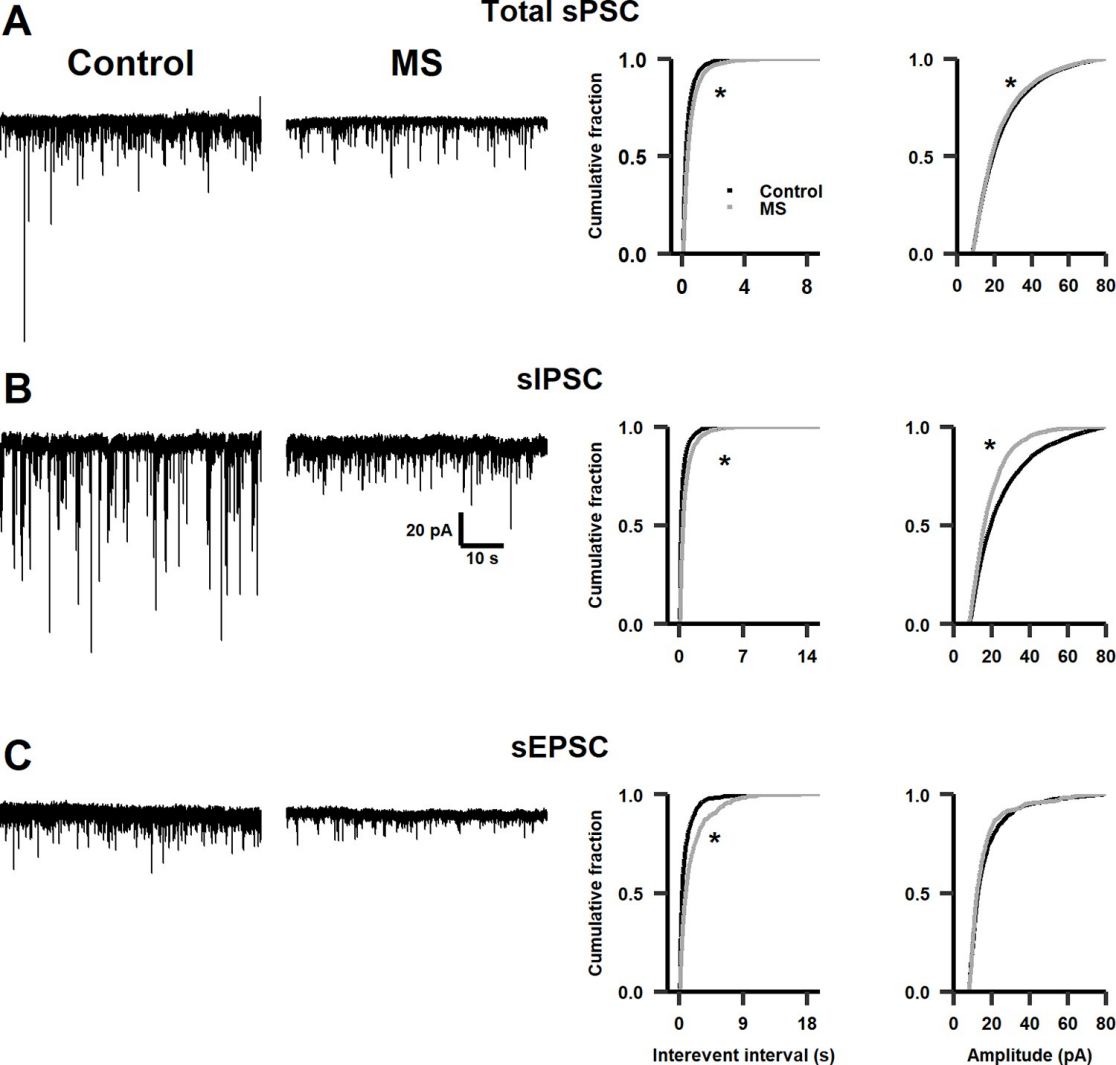
Effects of MS on synaptic activity in adult rats. **A.** Left panel: representative traces of total sPSCs in infralimbic cortex of control and adult MS-rats. Right panel: cumulative distribution plots of the interevent interval (Kolmogorov-Smirnov test, D = 0.22875, *p* < 0.001) and amplitude (Kolmogorov-Smirnov test, D = 0.032638, *p* < 0.01) of sPSCs (n = 18 cells, N = 11–12 rats). **B.** Similar as in (**A**) for sIPSCs (n = 10 cells, N = 7–9 rats, interevent interval Kolmogorov-Smirnov test, D = 0.34221, *p* < 0.001, amplitude Kolmogorov-Smirnov test D = 0.15967, *p* < 0.001). **C.** Similar as in (**A**) for sEPSCs (n = 8 cells, N = 8 rats, interevent interval Kolmogorov-Smirnov test, D = 0.24808, *p* < 0.001, amplitude Kolmogorov-Smirnov test D = 0.065556, *p* = 0.075). * *p* < 0.01.

Finally, in infralimbic neurons of adult MS-rats, the IEI of sPSCs increased, and then the frequency of synaptic events together with the amplitude of sPSCs decreased, compared to control rats (Fig 5A). After isolating sIPSCs, the IEI of sIPSCs increased, corresponding to a decrease in the frequency of sIPSCs in neurons of adult MS-rats, the amplitude of sPSCs also decreased (Fig 5B). Besides, the IEI of sEPSCs significantly increased in MS-rats, with no significant differences in their amplitude (Fig 5C). These results show that the infralimbic synaptic activity of MS-rats continued changing with age, decreasing both inhibitory and excitatory synaptic activity during adulthood, even if they were no longer exposed to any other stressful events.

**Fig 6:**
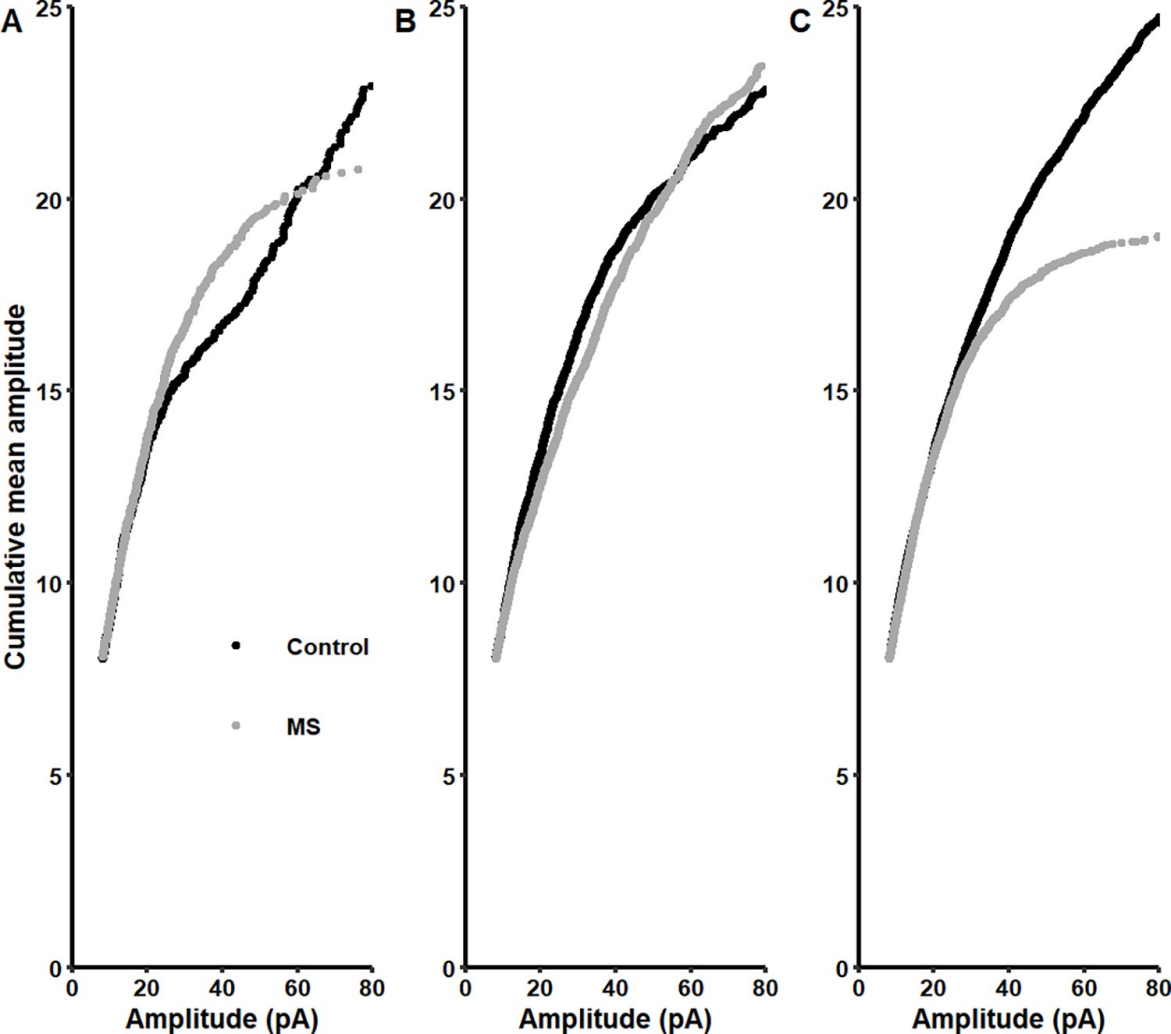
MS decreases the larger amplitude distribution in adult rats. Cumulative distributions of the mean amplitude of sIPSCs in infant **(A)**, adolescent **(B)** and adult rats **(C)**.

To further analyze the differences in amplitude of sIPSCs, we plotted the cumulative mean amplitude as a function of the amplitude to determine if there were changes in the population of small or large events [43, 48]. These plots suggest that a subtle increase of the sIPSCs occurred in the amplitude interval 30–60 pA in infant MS-rats (Fig 6A). For adolescent MS-rats, there was no clear differences in the distribution of the amplitude (Fig 6B). On the other hand, there was a marked decrease in the large amplitudes of sIPSCs (~40–80 pA) for adult MS-rats (Fig 6C). This decrease of large amplitudes is due to a change of multivesicular release from presynaptic terminals or to a reduction in the number of available receptors at postsynaptic neurons. Thus, we next analyzed the *q* of sIPSCs by fitting a Gaussian function (S4 Fig). However, the q value at the three ages did not show statistical differences between control and MS-rats. Thus, the changes obtained in amplitude could be attributable to a decrease of postsynaptic receptors.

### Membrane properties and intrinsic excitability

We next explored whether MS causes any alterations in the passive membrane properties and intrinsic excitability of infralimbic pyramidal neurons in infant, adolescent, and adult rats. We found that MS did not cause significant differences in any of the variables analyzed under current- clamp mode or changes in the AP profile (S1 Table). Similarly, no changes were found in the number of AP evoked by depolarizing current steps in MS-rats (S5 Fig).

## Discussion

Our study provides evidence of a physiological adaptation occurring in the infralimbic cortex of rats subjected to MS. Specifically, MS impacts the E/I balance in deeper layers of the infralimbic cortex, shifting toward excitation during infancy; this balance changes towards inhibition in adolescence; and then both excitation and inhibition decrease in adulthood.

Although the rodent model of MS has been related to depressive- and anxiety-like behaviors [13, 18, 49], other studies have reported the absence of these behavioral effects [16–18], indicating that in some cases the subjects may present a resilient profile. However, the underlying physiological mechanism related to this resiliency is unknown.

We consider that the protocol used in the present work is consistent with moderate MS stress. Its short duration conforms with protective or inoculatory effects in rodents subjected to mild and moderate MS that resulted in proactive behaviors indicative of resilience to stress [16, 19, 20]. Thus, adolescent, and adult MS-rats did not exhibit anxiety- and depression-like behavior in the different behavioral tests, similarly as in other work, even with prolonged MS [17, 18]. This lack of responses may be associated with resilience to early life stress. In contrast and with prolonged MS, opposing behavioral results were obtained in adolescent MS-rats, however similar in adult MS-rats [13].

With this in mind, we suggest that the E/I imbalance reported here could be the result of compensatory mechanisms protecting the brain against potential deleterious effects in individuals subjected to ELS in the form of moderate MS, resulting in the lack of behavioral effects.

### Maternal separation increases glutamate levels in the infralimbic cortex of infant resilient rats

An E/I balance is crucial to maintain an optimal tuning of the cortical network [50, 51]. Changes in the glutamatergic system have been related to stress and they are opposite depending on the exposure time to stress; acute stress increases glutamatergic activity while chronic stress decreases it [27, 52, 53]. It is important to keep this in mind because acute stress has been related to memory improvement [54–56], whereas chronic stress has been linked to psychiatric diseases [27, 57]. Here we found that, in infant MS-rats, the frequency of sEPSCs increased in infralimbic cortex neurons, which can be related to an increased glutamate release from neurons contacting these infralimbic cells. This is the first report in which synaptic activity has been recorded one day after the last MS at an infant age. Interestingly, this effect on synaptic activity resembles the acute stress that induces plastic changes to improve cognitive flexibility [58]. Our results support the idea that an increased E/I ratio in frontal regions during an early life period is relevant for proficient functioning of cortical circuits [59, 60]. Moderate MS protects from deleterious effect of chronic social defeat stress in adult mice by increasing miniature EPSCs in the basolateral amygdala [16]. Recently, however, it was found that MS decreases the expression of the BDNF protein and GAD65 mRNA levels in the mPFC during P7 and P21, respectively, indicating that those changes may cause an E/I imbalance in the mPFC related to a social recognition deficit [10, 61]. Although more studies are required to elucidate the role of glutamate in ELS, these data suggest that glutamate levels in the infralimbic cortex might serve as a biomarker of stress resilience.

### Maternal separation modifies the GABAergic system in the infralimbic cortex of adolescent resilient rats

Information regarding the effects of early stress in mPFC in adolescent subjects is sparse, but in general, it agrees with the idea that early stress causes less inhibition in the mPFC and that is associated with anxiety-like behavior. For instance, in MS-rodents, the number of GABA_A_R, AMPAR, and density of interneurons decrease in the PFC and hippocampus [62, 63]. Recently, Chen and collaborators [13] studied in adolescent and adult male MS-rats the electrical characteristics in pyramidal neurons of deep layers in prelimbic cortex. Interestingly, they found that prelimbic neurons increased their intrinsic excitability and frequency of sEPSCs in adolescent MS-rats expressing anxiety-like behaviors and anhedonia [13]. Similarly, AMPAR subunits increase in pyramidal neurons from prelimbic layers II/III [6]; while the opposite occurs in synaptic excitability [49]. Although the behavior was not analyzed, this discrepancy could be due to differences of the stress model. In our study, we found a discrete increase in the frequency of sIPSCs in adolescent MS-rats, which did not express maladaptive behaviors. Likewise, in rats resilient to stress, the number of GABAergic neurons containing the neuropeptide Y enhanced in the whole mPFC, and the frequency of sIPSCs decreased in the anhedonic rats [64]. Possibly, compensatory mechanisms are triggered to avoid increase of excitatory activity leading to excitotoxicity [65, 66], similarly as in other neuronal disorders [67, 68]. Therefore, these mechanisms may promote adaptive states, allowing individuals to deal with adverse conditions, protecting for the development of anxiety- or depressive-like behaviors. That is the case of infralimbic neurons, in which intrinsic membrane properties and excitability did not change, presenting resilience against stress [21].

### Maternal separation hypoactivates the infralimbic cortex of adult resilient rats

In our study, we found that the frequency and amplitude of inhibitory synaptic activity, as well as the frequency of excitatory synaptic events, decreased in infralimbic neurons of adult MS-rats. However, in general terms, MS on adult subjects induces an E/I imbalance in the mPFC towards excitation, that is related to anxiety- and depressive-like behaviors [61, 62, 69, 70]. Other studies report contrasting results, which are related to the age at which the stressful events occurred [64, 71]. Moreover, there is a smaller number of inhibitory cells and synapses in the infralimbic cortex, associated with social deficit after MS [61]; unfortunately, we did not perform the social interaction test to compare these findings. In other studies, the activation of infralimbic cortex inhibits the dopaminergic pathways related to reward, thus causing anhedonia [72, 73]. Additionally, projections of the infralimbic cortex regulate the activity of the hypothalamus-pituitary-adrenal axis [74–76]; therefore, its activation may cause an increase of corticosterone release. Besides this, the hyperactivation of other downstream projections, such as the mPFC-amygdala pathway, are related to deleterious effects [77]. On the other hand, infralimbic cortex sends glutamatergic afferents to intercalated cells, which in turn inhibit amygdala output and fear expression [78, 79]. All this suggests that, depending on the downstream projection, the inhibition of infralimbic cortex has positive or negative effects on behavior. Additionally, we cannot exclude that other maladaptative behaviors associated with MS and the functioning of infralimbic cortex are related to our results, such as alterations in fear extinction, aggressive behavior, object recognition and even alcoholism, which were previously related to MS [80, 81]. Hence, further studies are needed to elucidate this, particularly during infancy and adolescence.

Nevertheless, modulation of infralimbic pyramidal neurons by both excitatory and inhibitory synaptic inputs may be relevant to avoid the adverse effects of stress and lead to resilience mechanisms against MS stress. In this way, the integration of excitatory and inhibitory inputs in infralimbic neurons regulates their output signaling [82], together with the fact that infralimbic neurons project to areas associated with vulnerability or resilience to stress [83]. In this regard, in the actual study, we found that sEPSCs and sIPSCs in infralimbic neurons changed in MS-rats, depending on their age (infant, adolescent and adult). Thus, the frequency of sIPSCs increased and decreased in adolescent and adult MS-rats, respectively, compared with control rats. These changes, together with the absence of anxiety- and depressive-like behaviors could be associated with resilience to MS. Thus, it is likely that some of the infralimbic neurons in MS-rats that we recorded project to the dorsal raphe, a neuronal circuit involved with depression and stress resilience [84, 85]; and then, the increase in frequency of sIPSCs in infralimbic neurons would disinhibit 5-HT neurons, resulting in an increase of 5-HT release from dorsal raphe neurons that, in turn, may contribute to the lack of depressant-like behavior, according with the monoamine hypothesis of depression [86]. In contrast, the enhancement of neuronal excitation in infralimbic pyramidal neurons leads to anxiety-like behavior [87].

Furthermore, molecular changes in the structure of GABA_A_R have been proposed as one potential mediator of anxiety and depression [88–90]. Thus, it is remarkable that gene expression of GABA_A_R α1 subunit increases in the mPFC of mice subjected to chronic restraint stress [89]. Conversely, reduced expression of this subunit is associated with fewer anxious and depressive behaviors. For instance, when rats were handled 30 minutes and then subjected 2 hours to MS, thus reducing expression of the GABA_A_R α1 subunit, they showed more active behaviors in FST without changes in corticosterone levels [91]. In addition, the expression of the α2 subunit, related to anxiolytic effects and resilience to stress, changes during neurodevelopment, with higher levels in early postnatal days and lower levels in adulthood [88, 92–96]. Interestingly, in the present work, the number of large amplitudes of sIPSCs decreased, suggesting an altered expression of postsynaptic GABA_A_R α1 subunits, similarly as in another study [97], although the opposite can occur in other structures [98]. Thus, we suggest that, under our conditions, MS may cause changes in the α1/α2 subunit ratio that triggers a resilient effect; however, more experiments are required to confirm this result.

Finally, activity of the excitatory glutamatergic system increases in the rodent MS model. In this regard, glutamate may activate brain regions involved in symptoms of anxiety or depression, as mentioned before. Here, the frequency of sEPSC decreased in infralimbic neurons of adult MS-rats, suggesting reduction of presynaptic glutamate release in resilient MS-rats. This agrees with a previous study, in which evoked IPSCs were smaller and evoked eEPSCs were larger in infralimbic pyramidal neurons in mice with high anxiety-like behavior, and an AMPAR antagonist decreased this behavior [99]. Furthermore, in relation to our findings and with respect to depressive-like behavior, it has been proposed that the infralimbic cortex plays an essential role, given the antidepressant effect by the application of the glutamatergic antagonist ketamine in this brain region [100, 101].

## Conclusions

Overall, early-life stress in the form of MS causes resilience in infant, adolescent, and adult rats through dynamic changes in the E/I balance of infralimbic cortex. These changes are related to age-dependent differences in levels of glutamate release and GABA_A_R expression, with no effect in passive membrane properties and intrinsic neuronal excitability. The factors by which MS causes resilience and the specific pathways of the infralimbic cortex efferences related to anxiety- and depressive-like behaviors are not completely clear, and understanding these mechanisms will lead to a better treatment for these psychiatric diseases.

## Supporting information

S1 Fig Body and adrenal weights. **A.** Body weight of the animals at different ages (number of rats: P16 control/MS = 14/14, P22 control/MS = 24/22, P32 control/MS = 14/15, P60 control/MS = 17/15, linear mixed-effects models [age: F(3,25) = 1870.72, *p* < 0.001, group: F(1,99) = 2.2, *p* = 0.14, age x group: F(3,99) = 0.37, *p* = 0.78]). **B.** Relative weight of the adrenal cortex at different ages (number of rats: P16 control/MS = 13/13, P32 control/MS = 12/14, P62 control/MS = 11/8, linear mixed-effects models [age: F(2,16) = 7.4, *p* < 0.01, group: F(1,38) = 0.024, *p* = 0.88, age x group: F(2,38) = 1.62, *p* = 0.21]). Here and in all figures, data represent means ± SEM. Ctrl: control, MS: maternal separation.
(TIFF)

S2 Fig Locomotion in the open field test. MS did not impair locomotion of the rats in any age studied with the open field test (number of rats: juvenile control/MS = 15/16; adult control/MS = 15/13, linear mixed-effects models [age: F(1,26) = 44.76, *p* < 0.001, group: F(1,41) = 0.72, *p* = 0.4, age x group: F(1,41) = 0.16, *p* = 0.69]).
(TIFF)

S3 Fig Water intake. MS did not impair the total liquid intake of the rats in any age studied (number of rats: juvenile control/MS = 14/13; adult control/MS = 8/7, linear mixed-effects models [age: F(1,34) = 52.8, *p* < 0.001, group: F(1,26) = 0.11, *p* = 0.75, age x group: F(1,26) = 0.14, *p* = 0.71]).
(TIFF)

S4 Fig Maternal separation (MS) did not affect the quantal size (*q*). **A, C, E.** Representative amplitude distribution histograms for spontaneous inhibitory postsynaptic currents of one neuron from each group and age. Dotted lines represent the Gaussian fit. **B, D, F.** Comparison of the *q* values show no differences among groups at any age (weighted t-test, infants: t(6.4) = 0.88, *p* = 0.41; adolescents: t(8.5) = -0.86, *p* = 0.41; adults: t(18) = 0.09, p = 0.93). Black dots represent means ± SEM.
(TIFF)

S5 Fig Maternal separation (MS) did not affect the frequency of action potentials. Number of spikes as a function of depolarizing step currents. **A.** Infants linear mixed-effects models [group: F(1,80) = 0.93, *p* = 0.34, pulse x group: F(5,80) = 0.12, *p* = 0.99]). **B.** Adolescents linear mixed-effects models [group: F(1,127) = 5.44, *p* = 0.021, pulse x group: F(5,126) = 1.02, *p* = 0.41], followed by Bonferroni [*p* > 0.05 for each pulse]). **C.** Adults linear mixed-effects models [group: F(1,86) = 0.012, *p* = 0.91, pulse x group: F(5,85) = 0.073, *p* = 1]).
(TIFF)

S1 Table Passive membrane and intrinsic excitability properties of infralimbic pyramidal neurons. (DOCX)

S1 Data (XLSX)

S2 Data (XLSX)
