# Supplementary figures and images for: Maternal separation modifies spontaneous synaptic activity in the infralimbic cortex of stress-resilient male rats
Source: PLoS One. 2023 Nov 9;18(11):e0294151. doi: 10.1371/journal.pone.0294151 (PMC10635473; doi:10.1371/journal.pone.0294151)

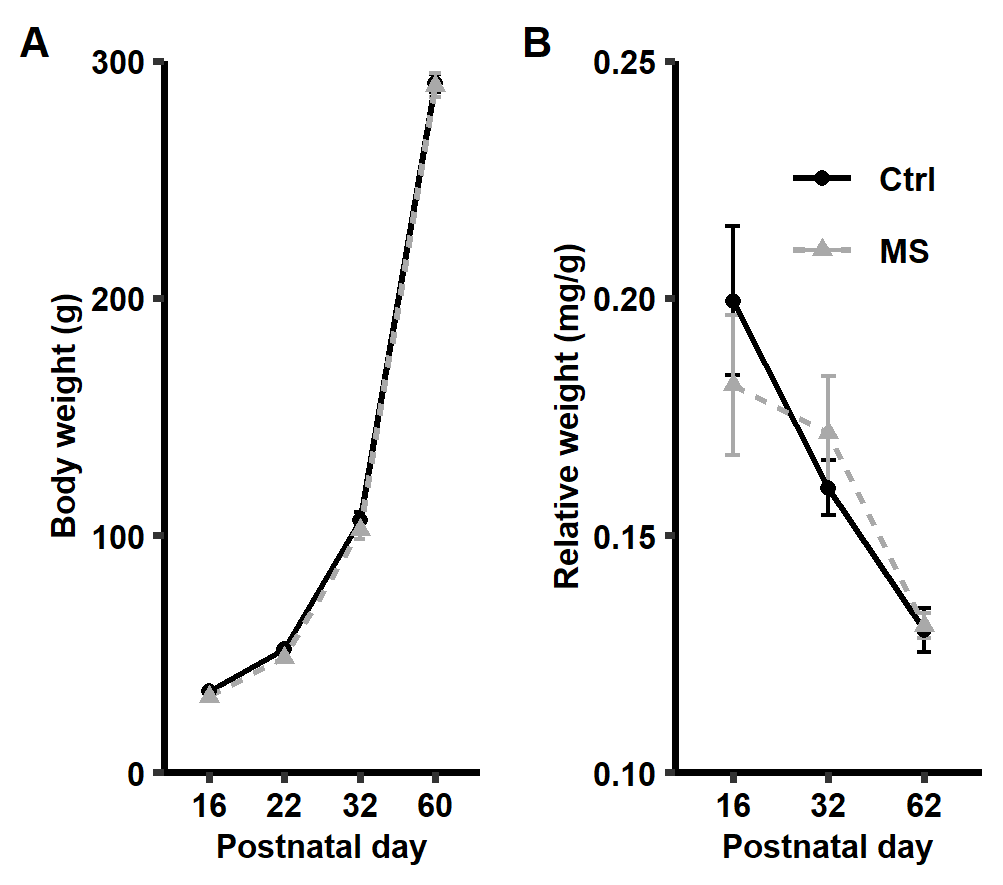

Supplement: S1 Fig — A. Body weight of the animals at different ages (number of rats: P16 control/MS = 14/14, P22 control/MS = 24/22, P32 control/MS = 14/15, P60 control/MS = 17/15, linear mixed-effects models [age: F(3,25) = 1870.72, p < 0.001, group: F(1,99) = 2.2, p = 0.14, age x group: F(3,99) = 0.37, p = 0.78]). B. Relative weight of the adrenal cortex at different ages (number of rats: P16 control/MS = 13/13, P32 control/MS = 12/14, P62 control/MS = 11/8, linear mixed-effects models [age: F(2,16) = 7.4, p < 0.01, group: F(1,38) = 0.024, p = 0.88, age x group: F(2,38) = 1.62, p = 0.21]). Here and in all figures, data represent means ± SEM. Ctrl: control, MS: maternal separation. (TIFF) [file pone.0294151.s001.tiff]

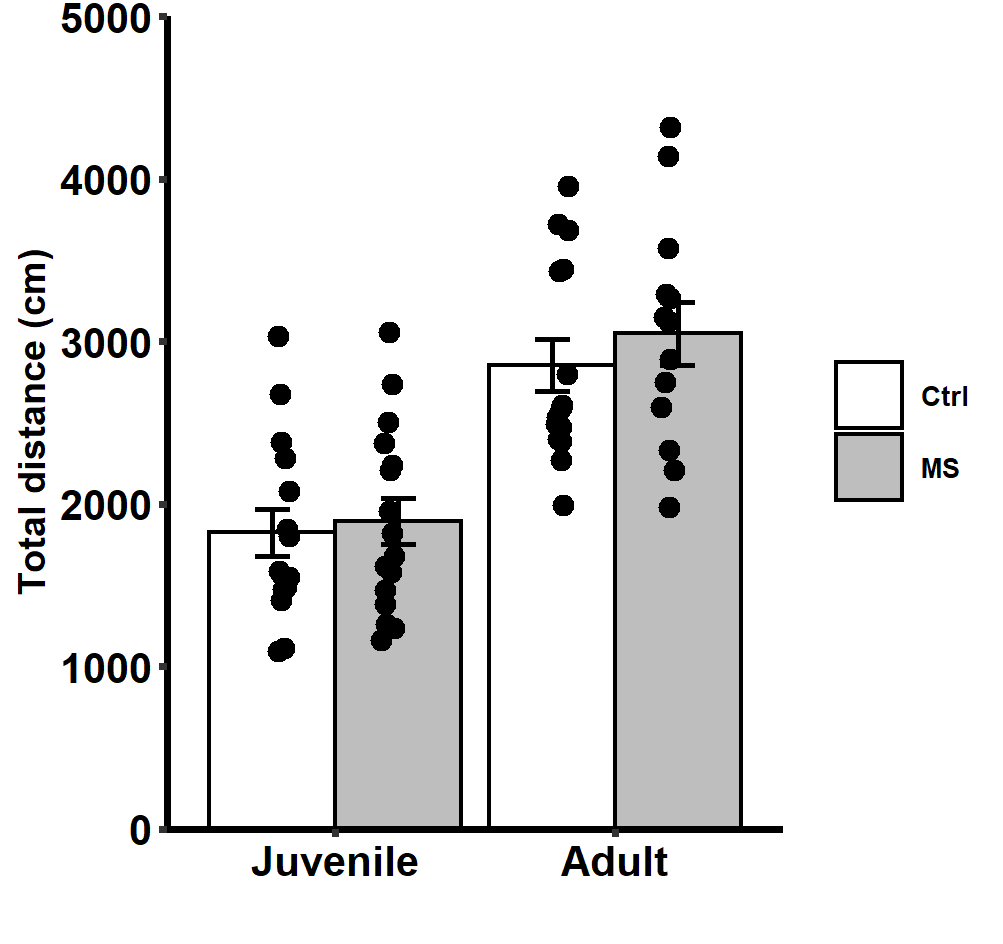

Supplement: S2 Fig — MS did not impair locomotion of the rats in any age studied with the open field test (number of rats: juvenile control/MS = 15/16; adult control/MS = 15/13, linear mixed-effects models [age: F(1,26) = 44.76, p < 0.001, group: F(1,41) = 0.72, p = 0.4, age x group: F(1,41) = 0.16, p = 0.69]). (TIFF) [file pone.0294151.s002.tiff]

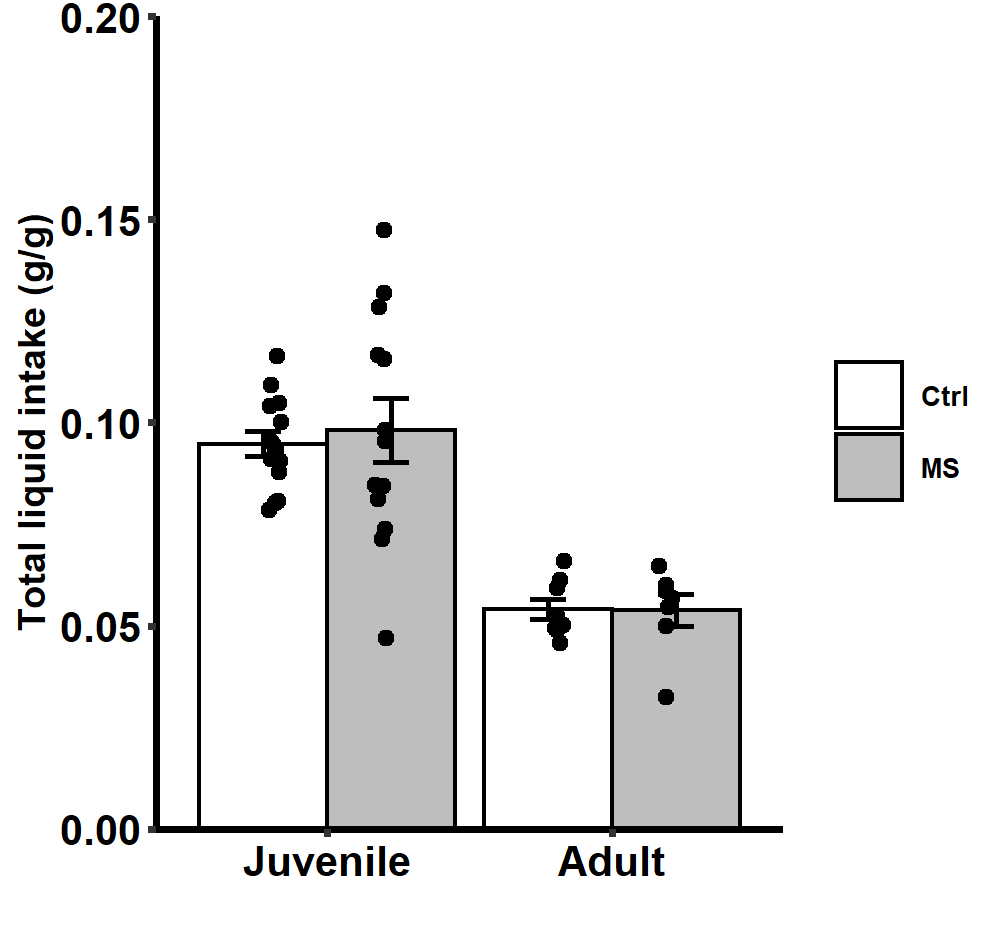

Supplement: S3 Fig — MS did not impair the total liquid intake of the rats in any age studied (number of rats: juvenile control/MS = 14/13; adult control/MS = 8/7, linear mixed-effects models [age: F(1,34) = 52.8, p < 0.001, group: F(1,26) = 0.11, p = 0.75, age x group: F(1,26) = 0.14, p = 0.71]). (TIFF) [file pone.0294151.s003.tiff]

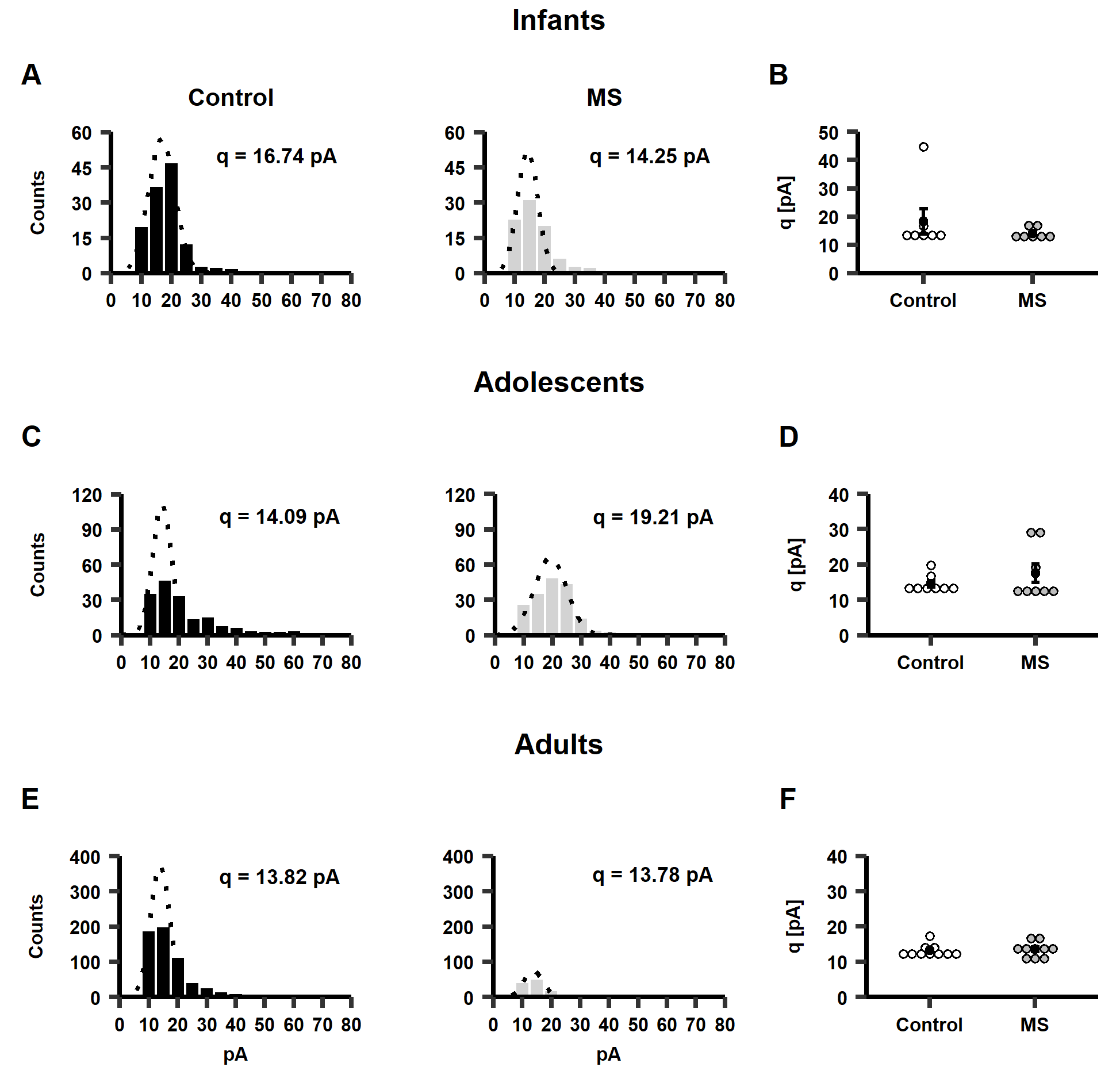

Supplement: S4 Fig — A, C, E. Representative amplitude distribution histograms for spontaneous inhibitory postsynaptic currents of one neuron from each group and age. Dotted lines represent the Gaussian fit. B, D, F. Comparison of the q values show no differences among groups at any age (weighted t-test, infants: t(6.4) = 0.88, p = 0.41; adolescents: t(8.5) = -0.86, p = 0.41; adults: t(18) = 0.09, p = 0.93). Black dots represent means ± SEM. (TIFF) [file pone.0294151.s004.tiff]

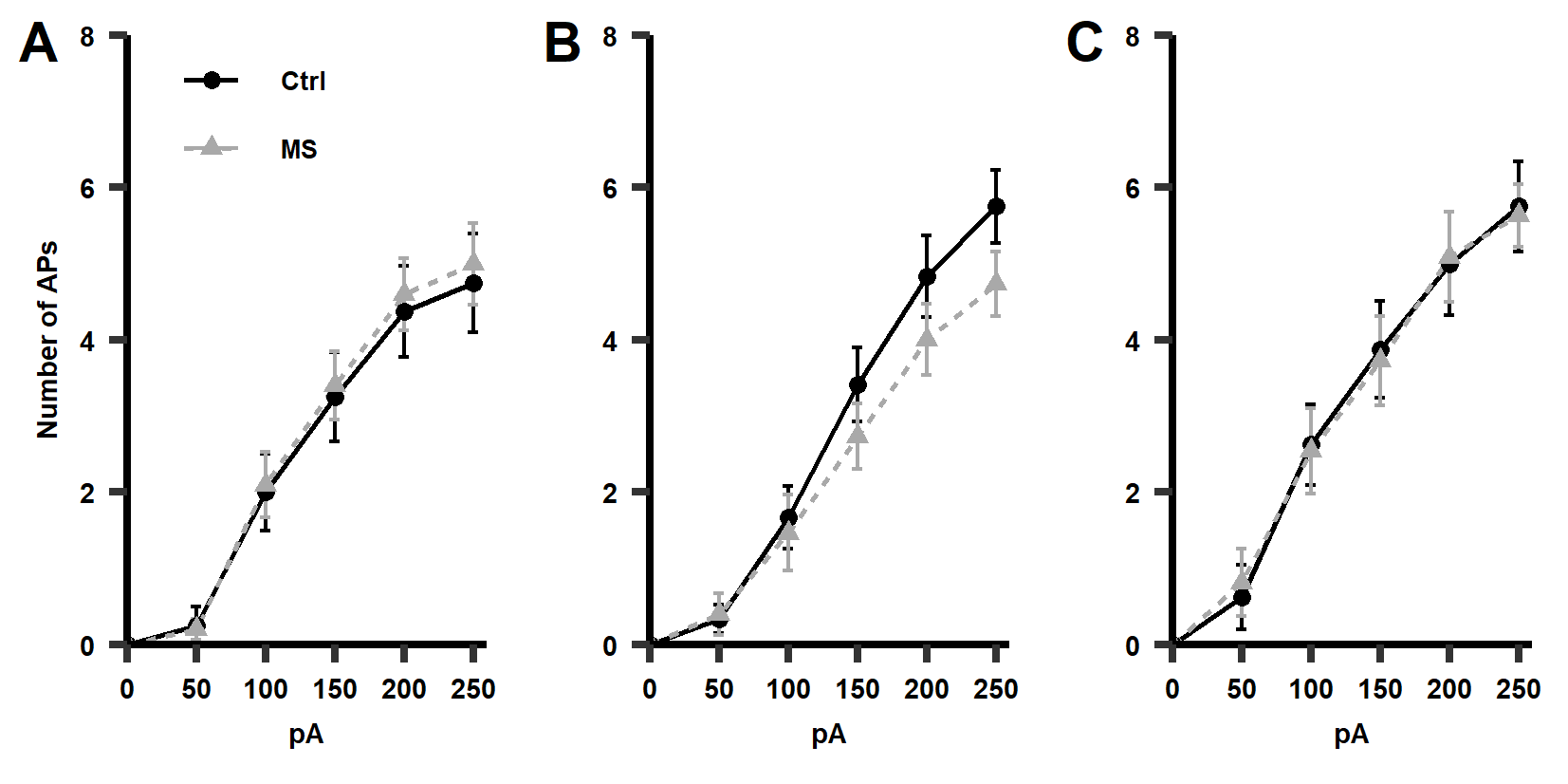

Supplement: S5 Fig — Number of spikes as a function of depolarizing step currents. A. Infants linear mixed-effects models [group: F(1,80) = 0.93, p = 0.34, pulse x group: F(5,80) = 0.12, p = 0.99]). B. Adolescents linear mixed-effects models [group: F(1,127) = 5.44, p = 0.021, pulse x group: F(5,126) = 1.02, p = 0.41], followed by Bonferroni [p > 0.05 for each pulse]). C. Adults linear mixed-effects models [group: F(1,86) = 0.012, p = 0.91, pulse x group: F(5,85) = 0.073, p = 1]). (TIFF) [file pone.0294151.s005.tiff]
