# Supplementary material for: Maternal separation modifies spontaneous synaptic activity in the infralimbic cortex of stress-resilient male rats
Source: PLoS One. 2023 Nov 9;18(11):e0294151. doi: 10.1371/journal.pone.0294151 (PMC10635473; doi:10.1371/journal.pone.0294151)
Supplement: S1 Table — (DOCX) [file pone.0294151.s006.docx]

|  | **Control** | | | **MS** | | |
| --- | --- | --- | --- | --- | --- | --- |
| **Property** | **Infant** | **Juvenile** | **Adult** | **Infant** | **Juvenile** | **Adult** |
| Membrane potential (mV) | -65.63 ± 1.31  (n = 8) | -65.25 ± 1.08  (n = 12) | -65.13 ± 1.46  (n = 8) | -64.10 ± 0.90  (n = 10) | -64.33 ± 1.14  (n = 15) | -66.09 ± 1.13  (n = 11) |
| AP amplitude (mV) | 58.26 ± 1.17  (n = 8) | 62.62 ± 3.75  (n = 12) | 63.93 ± 3.96  (n = 8) | 59.37 ± 2.71  (n = 10) | 64.15 ± 2.88  (n = 15) | 65.57 ± 1.98  (n = 11) |
| AP halfwidth (ms) | 2.36 ± 0.18  (n = 8) | 2.36 ± 0.11  (n = 12) | 2.51 ± 0.15  (n = 8) | 2.42 ± 0.09  (n = 10) | 2.11 ± 0.09  (n = 15) | 2.39 ± 0.13  (n = 11) |
| fAHP amplitude (mV) | -4.84 ± 1.70  (n = 8) | -1.7 ± 1.49  (n = 12) | -0.09 ± 0.98  (n = 8) | -4.43 ± 1.42  (n = 10) | -3.82 ± 1.34  (n = 15) | -3.82 ± 1.79  (n = 11) |
| AP threshold (mV) | -44.80 ± 2.15  (n = 8) | -46.40 ± 1.75  (n = 12) | -49.33 ± 1.72  (n = 8) | -42.95 ± 1.83  (n = 10) | -45.85 ± 1.85  (n = 15) | -45.11 ± 1.36  (n = 11) |
| dv/dt max (mV ms-1) | 82.25 ± 4.86  (n = 8) | 95.83 ± 9.07  (n = 12) | 91.26 ± 6.08  (n = 8) | 88.02 ± 9.22  (n = 10) | 100.2 ± 9.19  (n = 15) | 98.08 ± 6.78  (n = 11) |
| dv/dt min (mV ms-1) | -26.87 ± 2  (n = 8) | -28.57 ± 2.39  (n = 12) | -27.12 ± 1.84  (n = 8) | -25.77 ± 1.99  (n = 10) | -34.36 ± 2.41  (n = 15) | -30.01 ± 2.42  (n = 11) |
| Resistance (MΩ) | 212.3 ± 21.85  (n = 8) | 158.2 ± 15.90  (n = 12) | 135.6 ± 19.09  (n = 8) | 203.6 ± 20.47  (n = 10) | 157.5 ± 12.98  (n = 15) | 191.8 ± 19.52  (n = 11) |
| Instantaneous frequency (Hz) | 0.0182 ± 0.0039  (n = 8) | 0.0327 ± 0.0093  (n = 12) | 0.0237 ± 0.0065  (n = 8) | 0.0164 ± 0.0029  (n = 10) | 0.0278 ± 0.0067  (n = 15) | 0.0308 ± 0.0086  (n = 11) |
| ISI ratio | 2.69 ± 0.51  (n = 6) | 3.21 ± 0.29  (n = 9) | 2.86 ± 0.57  (n = 6) | 2.36 ± 0.25  (n = 8) | 3.01± 0.54  (n = 8) | 2.95 ± 0.49  (n = 9) |
| Sag ratio | 0.0297 ± 0.005  (n = 8) | 0.0401 ± 0.0051  (n = 12) | 0.0374 ± 0.0059  (n = 8) | 0.0413 ± 0.0072  (n = 10) | 0.0360 ± 0.0036  (n = 15) | 0.0362 ± 0.001  (n = 11) |

Linear mixed-effects models. Data represent means ± SEM. AP = action potential, fAHP = fast after hyperpolarization, ISI = interspike interval, MS = maternal separation, n = number of cells.
